# Virtual Reality Animal Rescue World: Pediatric virtual reality analgesia during just noticeable pressure pain in children aged 2–10 years old (crossover design)

**DOI:** 10.3389/fpsyg.2022.963765

**Published:** 2022-10-28

**Authors:** Taima Alrimy, Wadee Alhalabi, Areej A. Malibari, Fatma Salih Alzahrani, Sharifah Alrajhi, Mohammed Alhalabi, Hunter G. Hoffman

**Affiliations:** ^1^Department of Computer Science, Faculty of Computing and Information Technology, King Abdulaziz University, Jeddah, Saudi Arabia; ^2^Immersive Virtual Reality Research Group, King Abdulaziz University, Jeddah, Saudi Arabia; ^3^Department of Computer Science, School of Engineering, Computing and Informatics, Dar Al-Hekma University, Jeddah, Saudi Arabia; ^4^Department of Paediatric, King Abdulaziz University, Jeddah, Saudi Arabia; ^5^Department of Statistics, Faculty of Science, King Abdulaziz University, Jeddah, Saudi Arabia; ^6^Department of Mechanical Engineering, University of Washington, Seattle, WA, United States

**Keywords:** pain, analgesia, virtual reality, pediatric, non-pharmacologic, pain management

## Abstract

**Background and aims:**

Excessive pain during medical procedures is a worldwide medical problem. Most scald burns occur in children under 6, who are often undermedicated. Adjunctive Virtual Reality (VR) distraction has been shown to reduce pain in children aged 6–17, but little is known about VR analgesia in young children. This study tests whether desktop VR (VR Animal Rescue World) can reduce the just noticeable pressure pain of children aged 2–10.

**Methods:**

A within-subject repeated measures design was used. With treatment order randomized, each healthy volunteer pediatric participant underwent brief cutaneous pressure stimuli under three conditions: (1) no distraction, (2) a verbal color naming task (no VR), and (3) a large TV-based desktop VR distraction. A hand-held Wagner pressure pain stimulation device was used to generate just noticeable pain sensations. Participants indicated when a steadily increasing non-painful pressure stimulus first turned into a painful pressure sensation (just noticeable pain).

**Results:**

A total of 40 healthy children participated (43% aged 2–5 years; and 57% aged 6–10 years). Compared to the no distraction condition, the 40 children showed significant VR analgesia (i.e., a significant reduction in pain sensitivity during the VR Animal Rescue World condition), *t*(39) = 9.83, *p* < 0.001, SD = 6.24. VR was also significantly more effective at reducing pain sensitivity vs. an auditory color naming task, *t*(39) = 5.42, *p* < 0.001, SD = 5.94. The subset of children aged 2–5 showed significant reductions in pain during VR. Children under 6 showed greater sensitivity to pain during no distraction than children aged 6–10.

**Conclusion:**

During no distraction, children under 6 years old were significantly more sensitive to pain than children aged 6–10. Virtual reality (VR) significantly reduced the “just noticeable” pressure pain sensitivity of children in both age groups.

## Introduction

Excessive pain during medical procedures is a worldwide problem. Opioid analgesics are powerful, valuable, and often help reduce procedural pain, but have a number of side effects such as nausea, constipation, urinary retention, reduced respiration, and habituation, which reduce the dose levels prescribed to patients, and limit analgesic effectiveness (Cherny et al., 2001; Holtman and Jellish, 2012; Wasiak et al., 2014; Bittner et al., 2015; Retrouvey and Shahrokhi, 2015). Doctors are nervous about giving powerful opioid analgesics to children and are especially hesitant to give opioids and other pain medications to young children under the age of six. There may also continue to be some lingering misconceptions by some medical professionals about whether young children and infants can even feel pain (they can) (Wilson-Smith, 2011; Lea and Nichols, 2021).

For a number of reasons, due in part to the medical community’s current heavy reliance on pharmacologic analgesics alone, excessive pain during medical procedures is common for a wide range of medical procedures, and this is true for both adults, children and infants. In light of the current opioid overdose death crisis in the Western world (Chen et al., 2019) and increasingly strict federal regulation of prescription pain medications, a powerful non-pharmacologic analgesic that can be used in addition to, and in some cases, instead of analgesic pharmacology is a national priority (Keefe et al., 2018).

## Psychological pain interventions

The subjective experience of pain can be influenced by a number of psychological factors (Melzack and Wall, 1965). Anxiety (Ploghaus et al., 2001), the anticipation of pain (Fields, 2018), memories of previous painful experiences (Noel et al., 2015), catastrophizing (Petrini and Arendt-Nielsen, 2020), direction of attention (Birnie et al., 2017) and other psychological factors can increase how much pain a person experiences from any given noxious stimulus. Fortunately, because pain has a strong psychological component, psychological treatments can thus help reduce anxiety and other unhelpful psychological influences and can help reduce the intensity of pain that patients experience during medical procedures.

Conventional distraction techniques such as talking to the patient or letting patients listen to music during medical procedures can help reduce the pain of children and infants (Birnie et al., 2017). Attention demanding color naming tasks such as the Stroop task have been shown to reduce pain and can reduce pain related brain activity (Bantick et al., 2002), but stronger, more effective distractions are needed (Bellieni et al., 2013).

## Virtual reality analgesia

Attention is important for pain perception (Melzack, 1993; Eccleston and Crombez, 1999). Where the patients’ attention is directed can influence how much pain they experience. For example, if a patient is watching a nurse perform burn wound care, the patient’s brain is receiving converging/consistent evidence from visual input (looking at their bloody unbandaged burn wound) and nociceptive input from their pain receptors in the skin near their burn wound care.

The use of immersive VR distraction as a non-drug pain control technique was introduced in the 1990s (e.g., Hoffman, 1998; Hoffman et al., 2000). The logic is as follows: Pain requires attentional resources. VR floods the sensory system with pleasant (non-pain-related) computer-generated information, reducing the amount of attention the patient’s brain has available to also process nociceptive signals coming into the brain from pain receptors. A number of studies have now shown that interacting with a computer generated world using VR goggles can reduce pain in children aged 6 and older (Hoffman et al., 2019, 2020) during burn wound care and burn-related physical therapy skin stretching exercises, and for a growing number of painful or stressful pediatric medical procedures such as venipuncture, and dental procedures (see Trost et al., 2021 for a brief review). However, despite encouraging empirical results, until recently, immersive VR has been too expensive and too technically demanding (difficult to use) for widespread acceptance into everyday medical practice.

Due to substantial recent technological advances, VR is proving to be an effective, low cost and low-risk adjunctive non-pharmacologic analgesic to help reduce acute pain of patients during painful medical procedures in children 6 years and older, adults, and the elderly (see Trost et al., 2021 for a brief review of the VR literature). The new VR systems are highly portable, DC-powered, inexpensive, and do not require a specialized technician. As a result, a growing number of hospitals are exploring the use of VR for a wide range of medical procedures (Trost et al., 2021), and the total number of PubMed-indexed publications on the topic of VR analgesia for burn injuries has increased more than 40% between April 2019 and April 2022.

Despite these technological and empirical advances, little is known about VR analgesia in children under 6 years of age, due in part to the limitations of traditional head-mounted VR displays (i.e., VR helmets are not designed for children under 6 years old). This gap in the scientific literature (the rare use of VR analgesia in children under 6 years of age) is especially unfortunate since most pediatric scald burns occur in children under six (American Burn Association [ABA], 2017) and because infants and toddlers burn more quickly and at lower temperatures than older children and adults (American Burn Association [ABA], 2018). Recently, researchers have begun to explore the use of projector-based VR for young children during burn wound care, with encouraging results (Khadra et al., 2020). In those studies, pre-distorted computer-generated image streams were digitally projected onto an immersive dome rear projection screen positioned near the patient. And children could interact with objects they see in the projector dome (no VR helmets used, Khadra et al., 2020).

Most pediatric VR analgesia studies to date have involved patients aged 6 and older, and have used immersive VR with head mounted VR goggles with “near eye” lenses. For example, in previous studies using fully immersive VR, participants could look around a virtual world, using head tracked head mounted VR goggles (or *via* mouse tracking in some studies, e.g., Hoffman et al., 2019), and could interact with objects in the virtual environment (VE) *via* a computer input device such as a computer mouse (e.g., Al-Ghamdi et al., 2020).

## Desktop virtual reality

“VR uses computer technology to create an interactive three dimensional virtual world in which a user or multiple users can experience a simulated environment. The level of immersion of VR simulations can vary from the use of less immersive desktop VR to the high immersive head-mounted display (HMD) VR.” (Liaw et al., 2022, p. 2, see also Li et al., 2020)

The software used in the current study, “VR Animal Rescue World,” is a VR world simulation of an outdoor world, custom designed for pain distraction of children during painful procedures. For the current study, we designed a 3D VR environment that is suitable for children aged 2 and older during painful stimulations. Although the software can also be used in fully immersive mode (with head tracking, hand controllers, and a VR helmet) we used the Desktop VR version in the current study, because of the young age of the children (see Khadra et al., 2020).

The current laboratory pain study is designed to test the analgesic effectiveness of our new Desktop VR system in healthy children aged 2–10, using a Quantitative Sensory Testing “just noticeable pain” pressure paradigm.

### Primary objective

The primary objective was to determine whether Desktop VR distraction can reduce children’s sensitivity to brief pressure pain stimuli compared to a plausible control condition (a “verbal only” color naming task) and compared to no distraction.

## Methodology

**FIGURE 1:**
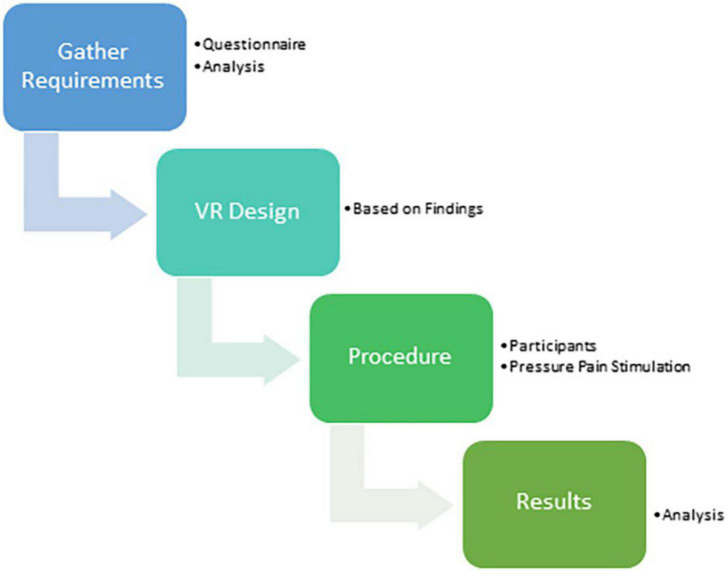
Methodology steps. Image by Taima Alrimy.

The main steps to achieve the goal of this research were as follows: A questionnaire was used to gather the needs and requirements of the children, to help design the VR environment and measure how much the VR system reduced pain. The methodology steps can be summarized in Figure 1.

## Requirements of the proposed virtual reality design

An online questionnaire used to help design VR Animal Rescue World was created using Google Forms. The questionnaire was distributed on social media -such as WhatsApp, Twitter, and Telegram- targeting parents or guardians of children between 0 and 5 years old. The main goal of the questionnaire was to illustrate the relationship between these children and technology, and how much they are exposed to the technology (what type of devices the children use, and how/when they use the devices). Also, to find out how familiar parents and children are with VR and what type of VR experiences children would likely prefer. The questionnaire has helped to understand the design requirements for the targeted age group (0–5 years) and their needs, and parent and patient recommendations. The design took into consideration children’s familiarity with TV screens and their love for cartoons, animals, and sounds. Also, because of their young age group and early cognitive development ability, large, simple virtual objects were used to encourage simple interaction. In addition, to help increase distraction, the virtual world is attractive in colors and sounds and designed to give users a sense of wonder and adventure, and is highly interactive (see also Al-Ghamdi et al., 2020; Hoffman, 2021). VR Animal Rescue World is designed to be very simple and easy for young users, in anticipation that they may be on pain medications and anticipating that it may be more difficult to interact with a virtual world when in pain.

## Virtual reality system design

**FIGURE 2:**
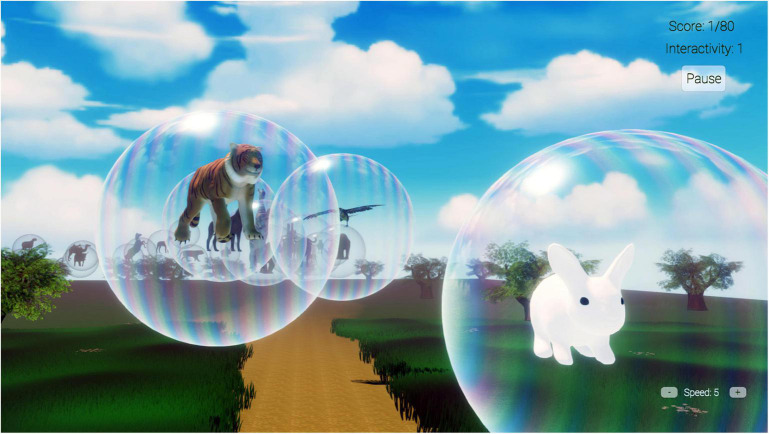
A screenshot of VR Animal Rescue World.

The current study tested a new desktop/TV screen-based VR world we named “VR Animal Rescue World,” custom designed and computer programmed by author TA for children aged 2–10. In VR Animal Rescue World, animals are trapped/imprisoned inside big transparent bubbles floating in the air in an outdoor nature scene, as displayed in Figure 2 (e.g., a tiger, a large cat, an elephant, a rabbit, and an eagle, etc., one bubble per animal). As the participant approached the nearest bubble, the animal trapped in that bubble made appropriate animal noises designed to indicate the animal wanted out of the bubble. The player clicked the bubble to break the bubble and let the animal float gently back to Earth, to freedom. The participant’s goal is to free the animals from the bubbles, so the virtual world is called Animal Rescue. The child’s viewpoint progressed along a pre-programmed spline path past a long series of floating bubbles. Each bubble had an animated animal inside. The participant could also interact with a saved animal on the ground; if touched by the cursor, the animal played its sound and glowed. The mixture of animal sounds and animated animal movements helps create a sense of presence and immersion in the virtual world. A bubble score and interactivity score were calculated to measure the player interaction.

## Software and materials

The VE was designed using the Unity^®^ game engine and visual studios for coding in the object-oriented c# programming language. In addition, the game shaders use Universal Render Pipeline (URP), which provides optimized graphics prebuilt by Unity^®^. The design runs on Windows^®^ 10 HP gaming laptop powered by a 2.80 GHz Intel^®^ Core COULDN’T i7-7700HQ CPU, 16GB of RAM, and an NVIDIA^®^ GeForce^®^ GTX 1050 Ti GPU with up to 8 GB of dedicated video memory graphics. In the current study, the VE is displayed on a TV device for screen-based 2D VR with a computer mouse input device so children could interact with the environment.

## The virtual environment flowchart

**FIGURE 3:**
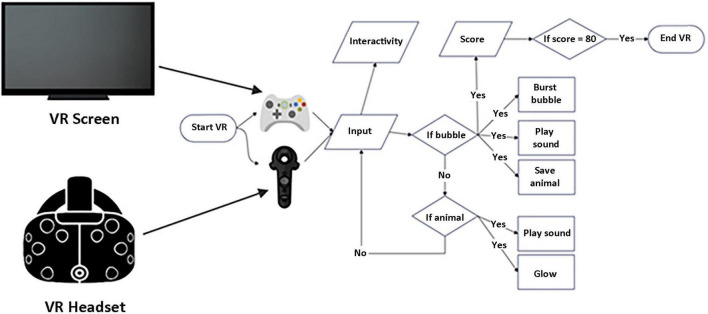
The VE flowchart.

The flow of the VE is presented in Figure 3: the VR experience starts with an automated move; each mouse click button is counted as interactivity. If a bubble is clicked, the bubble bursts with a sound, and the animal inside floats down to the ground/grass, and a score is calculated. If an animal on the ground is touched by a cursor, the animal glows with its sound.

Although the current study used the Desktop VR (no VR helmet), our “VR Animal Rescue World” is capable of fully immersive VR.

### Participants

Ethical approval to involve children’s participants was taken from the National Committee of Bio & Med. Ethics [NCBE] (Registration No. HA-02-J-008). A total of 40 healthy children aged 2–10 years old (43% aged 2–5 years; and 57% aged 6–10 years old) were recruited from a children center during a summer program, after their mother’s approval.

### Procedure

A Wagner FDX-25 (Wagner instruments, Greenwich, CT, United States) hand-held pressure pain stimulation device was used to generate a just noticeable pain sensation. The pressure algometer measures the linear force with a 1-cm^2^ round rubber tip. The rubber tip was placed on the volunteer’s body (arm or leg) and pressed on the skin, with a digital display of the amount of force exerted (we measured in units of Newtons). Volunteers were exposed to a discomforting sensation using the pressure pain stimulation system while interacting with the VR system to see how much the VR system provides a pain distraction. The volunteers were instructed to tell the researcher immediately when the pressure turned into a discomfort sensation. If so, the pressure was stopped, and the amount of pressure exerted was displayed and written down. Measurements (in Newton units) were taken three times per person, under 3 different treatment conditions (treatment order randomized) (1) while interacting with the VR system to see how much the VR design can provide pain distraction (2) when children sat in a normal relaxed position with no distraction, and finally, (3) when children performed a verbal color naming task in which they chose their favorite color (e.g., blue). The other person started naming different colors (red, green, black, etc.), and each time the participant heard their favorite color (e.g., blue), the participant called it out loud (e.g., “Blue”). The “just noticeable” pressure pain (pain threshold) measures were taken three times per participant, in a randomized order, using a brief 3-min washout period interstimulus interval. The same part of the body was used in each pressure pain stimulation, either the shoulder for three treatments or the leg for three treatments. The 3 min washout period used is based on Bisset et al., 2015. Bisset et al. (2015) compared the results of two pressure pain threshold protocols, and found no difference whether the interstimulus interval was 30 s or many min (Bisset et al., 2015, p 283). The maximum just noticeable pain pressure exerted (measured in Newtons) served as the primary dependent variable. We predicted that the desktop VR system would reduce the pain sensitivity of young children compared to no distraction and compared to a traditional distraction (the auditory color naming task).

## Results

**FIGURE 4:**
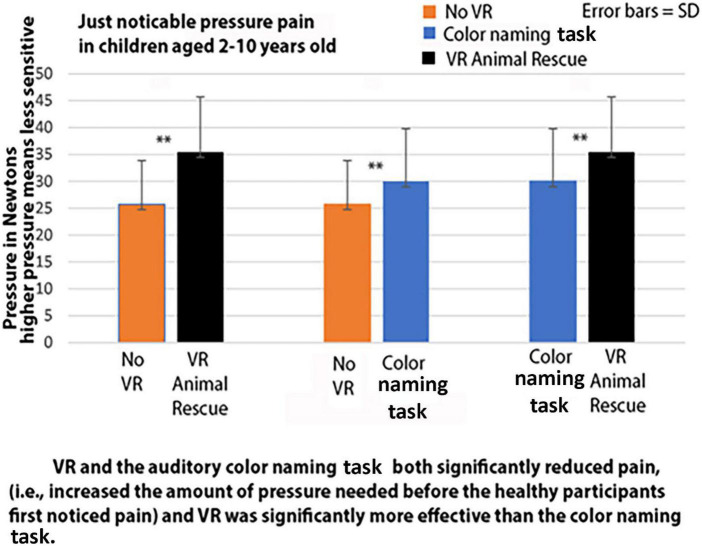
Virtual reality and the auditory color naming task both significantly reduced pain (i.e., increased the amount of pressure needed before the healthy participants first noticed pain) and VR was significantly more effective than the color naming task.

A within-subjects repeated measures ANOVA found a significant difference between the pressure exerted in the three treatment conditions, *F*(2,78) = 55.14, *p* < 0.001, η^2^ = 0.59. In *post hoc* paired *t*-tests, according to our Quantitative Sensory Testing, just noticeable pressure pain paradigm, as shown in Figure 4, compared to the no distraction condition, the 40 children showed significant VR analgesia (reduction in pain sensitivity during the VR Animal Rescue World condition). Specifically, using paired *t*-tests, VR significantly increased the amount of pressure needed before healthy children first noticed pain, *t*(39) = 9.83, *p* < 0.001, SD = 6.24. And VR also significantly reduced pain sensitivity compared to an auditory color naming task *t*(39) = 5.42, *p* < 0.001, SD = 5.94. And compared to no VR, the color naming task also reduced pain sensitivity, *t*(39) = 5.06, *p* < 0.001, SD = 5.35.

In *post hoc* comparisons, the subset of children aged 2–5 showed significant reductions in pain sensitivity during VR (see Tables 1, 2).

**TABLE 1:** Descriptive statistics.

		Mean pressure in Newtons	N	Std. deviation	Std. error mean
Pair 1	No distraction pressure	21.94	16	7.51	1.88
	VR pressure	33.11	16	8.85	2.21

Pair 2	No distraction pressure	21.94	16	7.52	1.88
	Color naming task pressure	25.37	16	6.77	1.69

Pair 3	Color naming task pressure	25.37	16	6.77	1.69
	VR pressure	33.11	16	8.85	2.21

For the subset of children aged 2–5, VR significantly reduced pain (i.e., increased the amount of pressure needed before the healthy participants first noticed pain).

**TABLE 2:** Paired samples *t*-tests.

	Mean difference scores	SD	SE mean	t	Df	Sig (2-tailed)
Pair 1: Pressure during no distraction vs. pressure during desktop VR	−11.16	6.42	1.61	6.95	15	<001

Pair 2: Pressure during no distraction vs. pressure during the color naming task.	−3.43	3.23	.81	4.24	15	<001

Pair 3: Pressure during the color naming task vs. pressure during desktop VR.	−7.74	5.41	1.35	5.71	15	<001

For the subset of children aged 2–5, VR significantly reduced pain (i.e., increased the amount of pressure needed before the healthy participants first noticed pain).

Similarly, in *post hoc* comparisons, the subset of children aged 6–10 showed significant reductions in pain sensitivity during VR (see Tables 3, 4).

**TABLE 3:** Descriptive statistics.

For the subset of children aged 6–10, VR significantly reduced pain (i.e., increased the amount of pressure needed before the healthy participants first noticed pain)	Mean pressure in Newtons	N	Std. deviation	Std. error mean
Pair 1: Pressure during no distraction vs. pressure during desktop VR	28.24	24	7.65	1.56
	36.97	24	11.03	2.25

Pair 2: Pressure during no distraction vs. pressure during color naming task	28.24	24	7.65	1.56
	33.10	24	10.38	2.12

Pair 3: Pressure during color naming task vs. pressure during desktop VR	33.10	24	10.38	2.12
	36.97	24	11.03	2.25

**TABLE 4:** Paired samples *t*-test.

For the subset of children aged 6–10, VR significantly reduced pain (i.e., increased the amount of pressure needed before the healthy participants first noticed pain)	Mean difference scores	Std. deviation	Std. error mean	t	df	Sig (2-tailed)
Pair 1: Pressure during no distraction vs. pressure during desktop VR	−8.73	6.06	1.24	7.06	23	<001

Pair 2: Pressure during no distraction vs. pressure during color naming task	−4.85	6.40	1.31	3.72	23	<005

Pair 3: Pressure during color naming task vs. pressure during desktop VR	−3.89	5.87	1.20	3.23	23	<005

According to a between groups analysis, just noticeable pain during no distraction occurred at a significantly greater pressure in children aged 6–10 than in children aged 2–5. In other words, children under 6 showed greater sensitivity to pain during no distraction (see Table 5).

According to a between groups analysis, just noticeable pain during no distraction occurred at a significantly greater pressure in children aged 6–10 than in children aged 2–5. In other words, children under 6 showed greater sensitivity to pain during no distraction (see Table 5).

**TABLE 5:** Descriptives.

	N	Mean	Std. deviation	Std. error mean
2–5 years	16	21.94	7.51	1.88
6–10 years	24	28.24	7.65	1.56
Total	40	25.72	8.12	1.28

Pressure exerted during no VR for children aged 2–5 vs. children aged 6–10 years old. According to a between groups analysis, just noticeable pain during no distraction occurred at a significantly greater pressure in children aged 6–10 than in children aged 2–5. In other words, children under 6 showed greater sensitivity to pain during no distraction. Between groups ANOVA, *F*(1,38) = 6.60, *p* = 0.01, MSW = 57.71.

## Discussion

During no VR, children under 6 years old were significantly more sensitive to pressure pain than children aged 6–10. Contrary to persisting misconceptions that young children do not feel pain, and thus the erroneous belief that young children do not need analgesia (Roofthooft et al., 2014; Goubert and Friedrichsdorf, 2021; Lea and Nichols, 2021), the current study showed that children under 6 years old are significantly more sensitive to pressure pain than children aged 6–10. This is another reason adjunctive analgesia customized for children under 6 years old is needed. Our new desktop VR Animal Rescue World analgesia system significantly reduced the “just noticeable” pressure pain sensitivity of children, showing VR analgesia in children under 6 years old for the first time in a laboratory VR pressure pain study. Overall, compared to no VR, the color naming task significantly reduced pressure pain in children aged 2–10. Furthermore, compared to No VR, and compared to the color naming task, which served as an attention control condition (Aycock et al., 2018; Tock et al., 2022), VR was able to significantly reduce the pressure pain sensitivity of healthy young children aged 2–10 while the participants were in the VR Animal Rescue world specifically designed for young children.

These results add to a growing literature showing analgesic benefits of digital distractions. For example, a recent meta-analysis of 106 studies involving digital distraction pain reduction (e.g., VR and video games) used during common procedures (e.g., venipuncture, dental, and burn treatments) came to the following conclusion: “For painful procedures, digital distraction resulted in a modest but clinically important reduction in self-reported pain…” (Gates et al., 2020, p1).

In another study, commercially available Nintendo Wii video games proved valuable for increasing range of motion of children with burns during physical therapy range of motion exercises (aged 5–17 years old) (Parry et al., 2012, 2014).

One within-subject case study found much larger reduction in acute pain during burn wound care during immersive VR vs. burn wound care during a conventional console Nintendo video game (Mario Kart) (Hoffman et al., 2000).

One recent clinical study found that interacting with the computer generated world significantly increased VR analgesia (Xiang et al., 2021, see also Wender et al., 2009; Hoffman, 2021).

A recent review and meta-analysis by Sajeev et al. (2021) concluded that interactive video games appear to reduce children’s procedural pain and anxiety and also reduced caregivers’ procedural anxiety.

## Conclusion

### Limitations

This study has a number of limitations that should be taken into consideration when interpreting the results. One important limitation is that the current study did not include any psychological measures, such as measures of how present the participants felt in VR (e.g., Hoffman et al., 1998; Hoffman, 2021). Another limitation is that the pain levels involved in the current study are very mild, and the results may or may not generalize to clinical pediatric contexts involving more severe pain levels. Another limitation is that the duration of VR in the current study was very short. Whether VR Animal Rescue World continues to be effective at reducing pain for longer treatment durations (e.g., 20 min) and whether it continues to reduce pain when used repeatedly is unknown. Results from two new “near eye” VR systems found VR reduced pain for clinically meaningful durations and continued to reduce pain when used repeatedly (Hoffman et al., 2019, 2020).

### Implications

Not only do young children feel pain, the current results suggest that young children aged 2–5 are significantly more sensitive to pressure pain than children aged 6–10 (children aged 2–5 are especially vulnerable to pain). Another major finding of the current study is that children aged 2–5 show VR analgesia. The implications are that children under 5 need non-pharmacologic adjuncts, and desktop VR analgesia appears to reduce pain in young children.

### Directions for future research

Most pediatric scald burns occur in children under 6 years old, and yet children in this age bracket are also likely to be undermedicated, due to concerns about giving powerful opioid analgesics to young children. Despite increased availability and ease of use, VR distraction is rarely used with patients under 6 years old. The current preliminary results suggest that additional research is warranted on whether desktop VR can reduce the extreme pain levels experienced by children during burn wound care. If so, this could be especially valuable for children during burn wound care, who are often too young to wear a VR helmet (under six) and/or who have burns on their heads and/or face that make it challenging for them to wear a head mounted VR helmet. Most tools used to measure psychological constructs are not available for children under 6 years old. New tools to measure psychological constructs in young children are needed (e.g., parent ratings of whether the child is generally anxious and/or prone to catastrophizing). Quantitative sensory testing could be used in future studies to potentially predict which pediatric patients are going to need adjunctive non-pharmacologic analgesics (e.g., children with low pressure pain thresholds on brief laboratory measures of individual patient sensitivity to painful stimuli during No VR). Research exploring the mechanism(s) of how VR reduces pain and how to maximize pain reduction and a better understanding of individual differences in analgesia during VR is recommended.

## Data availability statement

The raw data supporting the conclusions of this article will be made available by the authors, without undue reservation.

## Ethics statement

The studies involving human participants were reviewed and approved by the Regional Research Ethics Committee, Registered at the National Committee of Bio & Med. Ethics [NCBE] (Registration No. HA-02-J-008). Written informed consent to participate in this study was provided by the participants’ legal guardian/next of kin.

## Author contributions

All authors listed have made a substantial, direct, and intellectual contribution to the work, and approved it for publication.
